# Measuring Radiation Toxicity Using Circulating Cell-Free DNA in Prostate Cancer Patients

**DOI:** 10.14338/IJPT-D-21-00008

**Published:** 2021-07-27

**Authors:** Natalie A. Lockney, Randal H. Henderson, Steven G. Swarts, Zhenhuan Zhang, Bingrong Zhang, Jennifer Li, Robert A. Zlotecki, Christopher G. Morris, Katherine A. Casey-Sawicki, Paul G. Okunieff

**Affiliations:** Department of Radiation Oncology, University of Florida College of Medicine, Gainesville and Jacksonville, FL, USA

**Keywords:** biomarker, circulating DNA, cell-free DNA, protons, intensity modulated radiation therapy, prostate cancer, radiation toxicity

## Abstract

**Background:**

After radiation therapy (RT), circulating plasma cell-free DNA (cfDNA) released in response to RT damage to tissue can be measured within hours. We examined for a correlation between cfDNA measured during the first week of therapy and early and late gastrointestinal (GI) and genitourinary (GU) toxicity.

**Material and Methods:**

Patients were eligible for enrollment if they planned to receive proton or photon RT for nonmetastatic prostate cancer in the setting of an intact prostate or after prostatectomy. Blood was collected before treatment and on sequential treatment days for the first full week of therapy. Toxicity assessments were performed at baseline, weekly during RT, and 6 months and 12 months after RT. Data were analyzed to examine correlations among patient-reported GI and GU toxicities.

**Results:**

Fifty-four patients were evaluable for this study. Four (7%) and 3 (6%) patients experienced acute and late grade 2 GI toxicity, respectively. Twenty-two (41%) and 18 (35%) patients experienced acute and late grade 2 GU toxicity, respectively. No patients developed grade 3 or higher toxicity. Grade 2 acute GI toxicity, but not grade 2 acute GU toxicity, was significantly correlated with pre-RT cfDNA levels and on all days 1, 2, 3, 4, and 5 of RT (*P* < .005). Grade 2 late GI toxicity, but not GU toxicity, was significantly correlated with pre-RT cfDNA levels (*P* = .021).

**Conclusions:**

Based on this preliminary study, cfDNA levels can potentially predict the subset of patients destined to develop GI toxicity during prostate cancer treatment. Given that the toxicity profiles of the various fractionations and modalities are highly similar, the data support the expectation that cfDNA could provide a biological estimate to complement the dose-volume histogram. A test of this hypothesis is under evaluation in a National Cancer Institute–funded multi-institutional study.

## Introduction

Lack of a tool to predict radiation therapy (RT) toxicity is an important limitation in personalized care, as there are known variations in patients' normal tissue sensitivities to radiation [1–3]. Currently, radiation treatment regimens are based on population statistics aimed at achieving high cure rates with tolerable toxicity. Quantitative Analysis of Normal Tissue Effects in the Clinic (QUANTEC) or similar data that summarize the dose-volume relationships with toxicity risk [4] recognize interpatient variability and document the known relationship that exists between fraction dose and total dose with toxicity. While helpful, this approach is inadequate for personalized care as it does not differentiate the patients with intrinsic radiation sensitivity or the role of chemotherapy or intercurrent disease. A biomarker-based test that helps identify the more radiosensitive (or radiation tolerant) patients in a population would have many uses. Such a test would allow dose escalation to tumors in less sensitive patients, aid in the development of radiotoxicity mitigators for all patients, and reduce overtreatment risk for sensitive patients.

The QuantiDNA RadTox System (DiaCarta, Richmond, CA) is a patented technology (8,404,444: method for predicting the level of damage to cells by measuring free circulating arthrobacter luteus (Alu nucleic acid), which measures tissue damage shortly after RT exposure [5, 6]. The method is a simple measurement of total DNA in the circulation, using Alu retrotransposons as the DNA marker. This abundant repeat sequence accounts for at least 10% of the whole genome normally confined within cells; an estimated million copies of Alu are released per cell killed by irradiation. When most of the tissue exposure is normal tissue (not tumor), the circulating cell-free DNA (cfDNA) is an indicator of normal tissue toxicity; when most of the tissue exposure is treatment volume and tumor (such as with stereotactic ablative RT), it is an indicator of tumor response.

We previously reported data from a phase I/II clinical protocol demonstrating that RadTox cfDNA levels measured in patients undergoing RT for prostate cancer were correlated with body integral dose and detected the differential dosimetry of protons and photons [7]. In the current article, we report a secondary objective: To determine if plasma cfDNA measured early during an RT course is higher in patients who experience acute or late grade 2 or higher RT toxicity.

## Material and Methods

### Study Design and Patient Selection

Institutional review board approval for this phase I/II clinical protocol was obtained, and patients consented to participate. The study was conducted at 2 facilities at the University of Florida (UF): the UF Proton Therapy Institute in Jacksonville and the UF Health Shands Hospital in Gainesville. Patient inclusion and exclusion criteria and RT delivery details have previously been described [7].

Patients underwent pre-RT collection of plasma (20 mL) and 5 additional daily plasma collections (10 mL each) beginning 24 ± 4 hours after RT initiation to be used for circulating cfDNA measurement using the RadTox assay, further detailed later in this article. Radiation treatment plan details were recorded. The primary study objective was to determine if body integral dose is associated with RadTox and is reported separately [7]. A secondary objective was to determine if tissue damage measured using circulating cfDNA with the RadTox assay during treatment correlates with RT acute or chronic toxicity scoring. Patient-reported genitourinary (GU), gastrointestinal (GI), and other general toxicities (ie, fatigue, dermatitis) were recorded before treatment, weekly during treatment, and after treatment at 6 months and 1 year. Comparison of post-RT cfDNA levels and grade 2 or higher GI toxicity was planned before accrual. Patient-reported outcomes were captured via administration of the VisionTree Optimal Care (VisionTree Software, Inc, San Diego, California) survey, which is a patient-facing Common Terminology Criteria for Adverse Events (CTCAE, version 4.0, National Cancer Institute, Bethesda, Maryland) tool. Personnel involved in assessing toxicities were not aware of cfDNA data at the time of toxicity assessment.

### Circulating cfDNA Measurement Using the RadTox Assay

Venous blood was collected into 10-mL BD Vacutainer Plastic Blood Collection tubes with K_2_EDTA: Hemogard Closure (Greiner Bio-One, Frickenhausen, Germany). Blood was collected at a pre-RT blood draw (20 mL) defined as occurring any time in the week preceding initiation of radiation. Further, daily blood draws of 10 mL occurred on 5 consecutive treatment days beginning 24 ± 4 hours after initiation of RT. Treatments were begun on Mondays, Tuesdays, or Wednesdays; hence, there was always a 24- and 48-hour specimen, and the last specimen was at 7 days. Blood specimens were kept at room temperature for no more than 1 hour or stored on ice or at 4°C. Plasma was transferred into 1-mL microcentrifuge tubes and frozen at –80°C within 4 hours of collection. Plasma specimens were then shipped in dry ice to DiaCarta, Inc., where the RadTox assay was performed to measure DNA concentration (ng DNA per mL of plasma).

### Statistical Analysis

JMP Pro version 13.0.0 was used for all statistical analyses (SAS Institute, Cary, North Carolina). Univariate analyses were conducted to assess the association of baseline and on-treatment RadTox scores with both selected baseline prognostic factors as well as acute and late treatment-related toxicities. For the analysis of RadTox scores as a function of baseline prognostic factors, an independent *t-*test was appropriate. However, the toxicity analyses required that RadTox scores be treated as the prognostic factor and toxicity as the event. Logistic regression was therefore the appropriate technique. Receiver-operator curves were derived from the sensitivity and specificity estimates from each logistic regression. The area under the curve (AUC) and estimated optimal RadTox cutoff derived from Youden's J index are provided.

## Results

### Patient and Tumor Characteristics

A total of 70 patients consented to participate in the study. There were 54 evaluable patients, as 16 patients withdrew or became ineligible (8 were treated elsewhere, 6 decided against the blood draws, 1 developed a metastasis, and 1 was hospitalized for a noncancer issue). The median patient age was 69.8 years (range = 52.3–85.2 years). Most patients (78%) received proton RT alone, while 12 patients (22%) received either photon IMRT alone or photon IMRT followed by a proton boost. Patients who received photon immune-modulated RT (IMRT) followed by a proton boost had not yet begun the proton boost by day 5 of blood draw and, therefore, were assigned the photon IMRT (x-ray) group throughout the analysis. The median prescribed RT dose was 78 Gy (range = 66.6–78 Gy). Nineteen patients (35%) received androgen deprivation therapy concurrently with RT. Only 6 patients (11%) had undergone prostatectomy, while the remaining had an intact prostate (89%). Ten patients (19%) received treatment to the pelvic lymph nodes in addition to the prostate or prostate bed ± seminal vesicles.

### Patient-Reported Acute Toxicity

Acute toxicity was reported for all 54 patients and is shown in **Table 1**. All patients experienced at least 1 acute grade 1 or higher toxicity, and 24 patients (44%) experienced at least 1 acute grade 2 toxicity. No patients experienced any acute grade 3 or higher toxicity.

**Table 1.** Patient-reported radiation toxicity.

	**Grade 0, No. (%)**	**Grade 1, No. (%)**	**Grade 2, No. (%)**	**Grade 3, No. (%)**
Acute toxicity (n=54)				
Gastrointestinal	19 (35.2)	31 (57.4)	4 (7.4)	0 (0.0)
Genitourinary	0 (0.0)	32 (59.3)	22 (40.7)	0 (0.0)
Other	3 (5.6)	48 (88.9)	3 (5.6)	0 (0.0)
Late toxicity (n=51)				
Gastrointestinal	33 (64.7)	15 (29.4)	3 (5.9)	0 (0.0)
Genitourinary	11 (21.6)	22 (43.1)	18 (35.3)	0 (0.0)
Other	21 (41.1)	28 (54.9)	2 (3.9)	0 (0.0)

Note: Grading per Common Terminology Criteria for Adverse Events, version 4.0.

**Figure 1.**
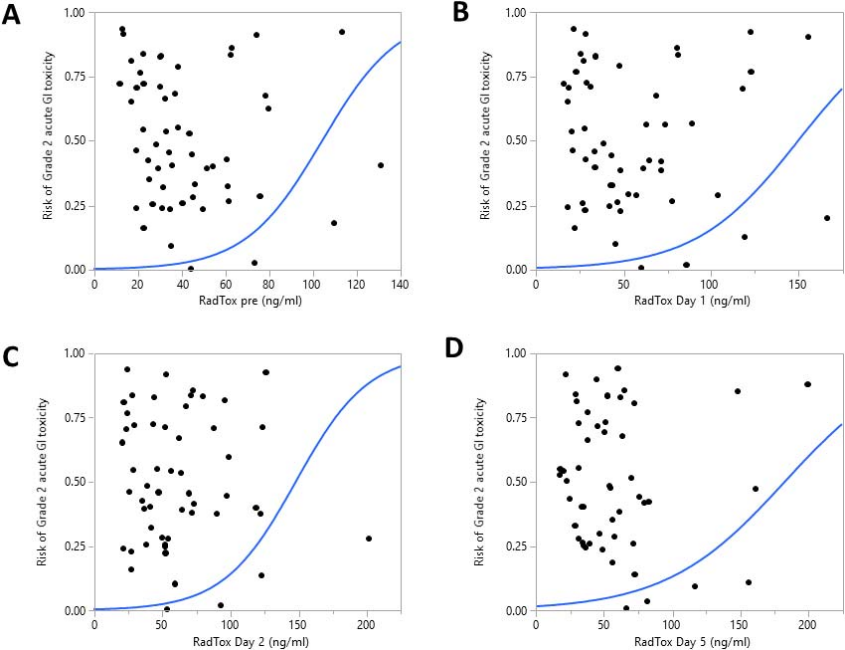
Logistic fit of grade 2 acute gastrointestinal (GI) toxicity by RadTox cfDNA levels measured (A) before radiation therapy (RT), (B) day 1 after RT, (C) day 2 after RT, and (D) day 5 after RT.

RadTox cfDNA levels measured on all days (pre-RT and days 1, 2, 3, 4, and 5 of RT) were significantly correlated with the presence of patient-reported grade 2 acute GI toxicity (*P* < .005), as shown in **Table 2** and **Figure 1**. However, RadTox levels were not significantly associated with patient-reported grade 2 acute GU toxicity.

**Table 2.** Association of RadTox score with patient-reported acute grade 2 radiation toxicity.

	**RadTox Measurement**					
	**Pre-RT**	**Day 1**	**Day 2**	**Day 3**	**Day 4**	**Day 5**
Acute grade 2 GI toxicity	.007^a^	.014^a^	.021^a^	.034^a^	.015^a^	.028^a^
Acute grade 2 GU toxicity	.985	.997	.685	.867	.718	.681

**Abbreviations:** RT, radiation therapy; GI, gastrointestinal; GU, genitourinary; cfDNA, cell-free DNA.

Note: RadTox was measured pre-RT and during the first 5 days of RT. Logistic regression analyses of RadTox level by presence or absence of development of acute GI or GU grade 2 toxicity were performed. Toxicity grading was per Common Terminology Criteria for Adverse Events version 4.0. We observed that higher RadTox cfDNA levels, measured pre-RT or any day during the first week of RT, corresponded with development of patient-reported acute grade 2 GI toxicity but not acute grade 2 GU toxicity.

a Significant *P* value.

### Patient-Reported Late Toxicity

**Figure 2.**
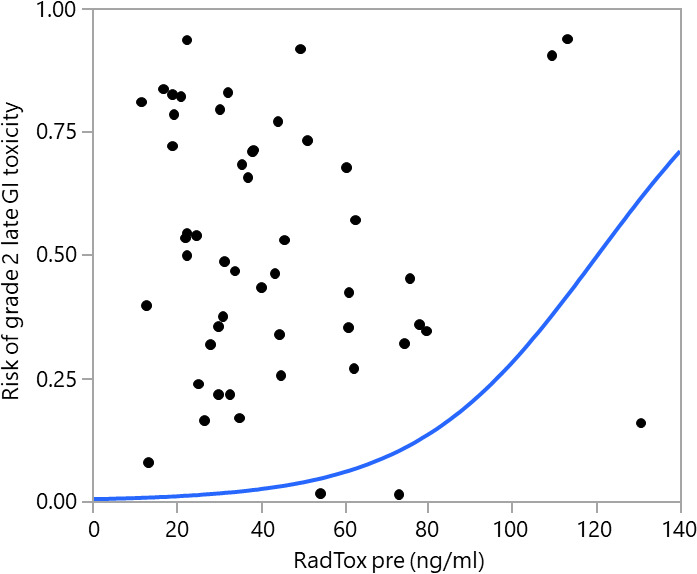
Logistic fit of grade 2 late gastrointestinal (GI) toxicity by RadTox cfDNA levels measured before radiation therapy (RT).

Late toxicity was reported for 51 patients and is summarized in **Table 1**. Forty-three patients (84%) experienced at least 1 late grade 1 or higher toxicity, and 14 patients (27%) experienced at least 1 late grade 2 toxicity. No patients experienced any late grade 3 or higher toxicity. RadTox cfDNA levels measured before RT, but not those measured after RT, were significantly correlated with patient-reported grade 2 GI late toxicity (*P* = .021), as shown in **Table 3** and **Figure 2**. RadTox levels were not significantly associated with patient-reported grade 2 late GU toxicity.

**Table 3.** Association of RadTox score with patient-reported late grade 2 radiation toxicity.

	**RadTox Measurement**					
	**Pre-RT**	**Day 1**	**Day 2**	**Day 3**	**Day 4**	**Day 5**
Late grade 2 GI toxicity	.021^a^	.196	.160	.156	.112	.145
Late grade 2 GU toxicity	.947	.601	.799	.737	.450	.788

**Abbreviations:** RT, radiation therapy; GI, gastrointestinal; GU, genitourinary; CTCAE, Common Terminology Criteria for Adverse Event.

Note: RadTox was measured pre-RT and during the first 5 days of RT. Logistic regression analyses of RadTox score by presence or absence of development of late GI or GU CTCAE grade 2 toxicity were performed. Toxicity grading was per CTCAE version 4.0. We observed that RadTox measured pre-RT was significantly correlated with development of patient-reported grade 2 late GI toxicity but not grade 2 GU toxicity.

a Significant *P* value.

### Receiver Operating Characteristics

**Figure 3.**
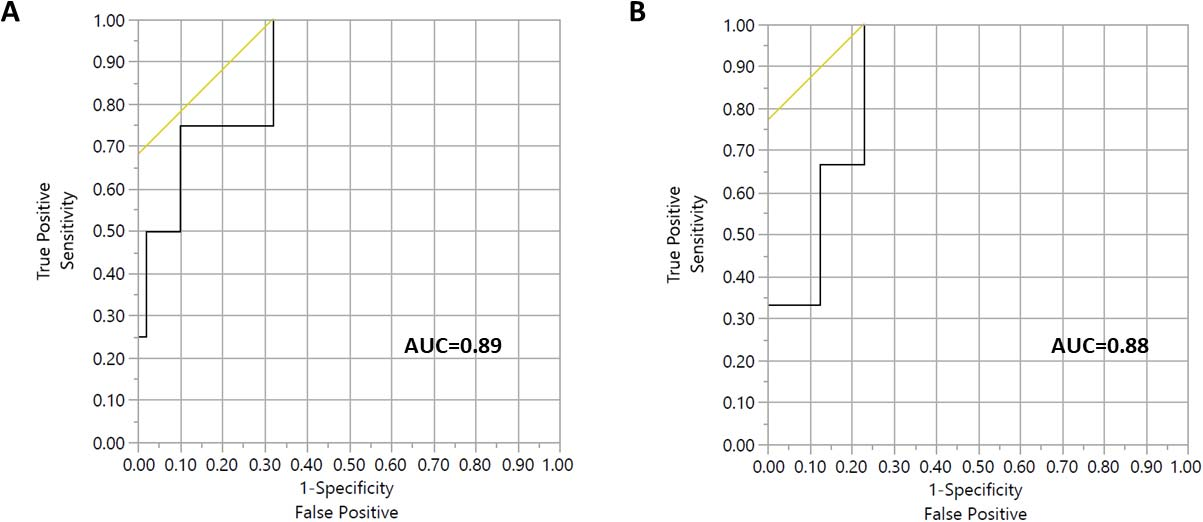
Receiver operating characteristics for pre–radiation therapy RadTox cfDNA levels with (A) acute grade 2 gastrointestinal (GI) toxicity and (B) late grade 2 GI toxicity.

As stated, RadTox cfDNA levels measured before RT were significantly associated with both acute (*P* = .009) and late grade 2 GI toxicity (*P* = .021). As shown in **Figure 3**, for pre-RT RadTox cfDNA levels with acute grade 2 GI toxicity, the AUC was 0.89, and the optimal cutoff was 44 ng/mL. For pre-RT RadTox levels with late grade 2 GI toxicity, the AUC was 0.88, and the optimal cutoff was 54 ng/mL.

## Discussion

Many researchers have investigated normal tissue response to RT in an effort to show correlation between clinically reported RT toxicities and in vitro cellular radiation sensitivity [1, 8–10]. However, limits of in vitro testing, such as variability in laboratory techniques, cell types, and inadequate clinical data, have accounted for inconsistent results. Gene-expression profiles have shown correlation with RT toxicity, and efforts have been made to develop predictive DNA and RNA signatures for toxicity [11–14]. There is a need for rapid assays for use in the clinical setting to predict an individual patient's radiosensitivity before or early in the RT course to allow time for interventions to tailor the patient's RT course—such as dose escalation or, for the radiosensitive patient, dose de-escalation or radiation mitigators. We demonstrated in this phase I/II clinical trial for patients with prostate cancer undergoing RT that measuring plasma cfDNA levels with the RadTox assay before RT or early in the course of RT predicted development of patient-reported acute and late grade 2 GI, but not GU, toxicity.

Higher RadTox levels measured before RT and on days 1–5 of RT were associated with the presence of patient-reported acute grade 2 GI toxicity. The data support the expectation that cfDNA levels may provide a biological estimate to complement the dose-volume histogram. Interestingly, the association of pre-RT cfDNA levels with both acute and late grade GI toxicity may allow identification of more highly radiosensitive patients before treatment initiation and should be a topic of further investigation. We have demonstrated in a preclinical mouse model that while use of lipopolysaccharide (LPS), as a surrogate for sepsis, did not raise baseline cfDNA levels alone, LPS combined with radiation resulted in a synergistic increase in cfDNA consistent with a synergistic toxicity [May 2008; PGO]. Therefore, other preexisting patient disease states may predispose patients to increased radiotoxicities.

The lack of statistical significance of GU toxicity is interesting. Modern radiation fields generally deliver high doses to only a small portion of the bowel, with most of the bowel receiving dose in the margin and low-dose wash [15–17]. Conversely, GU toxicity is generally related to base-of-bladder and urethral toxicity, and these regions are necessarily included in the full dose volume [18]. Thus, the dose to the urethra might be too high to prevent toxicity. Another potential explanation is the low total tissue volume of the posterior bladder wall and prostatic urethra, leading to only a small component of the total cfDNA. For distinguishing organs at risk, it will be important to try to develop organ-specific tests to supplement the total damage measurement provided by cfDNA.

Our study has several limitations. The evaluation of toxicity was a secondary aim, and the study was not powered to predict toxicity. There were few grade 2 GI toxicity events and no grade 3 events. The fact that a small study already showed significance for GI toxicity is promising, as this high degree of detection is needed for a personalized test. After radiation, cfDNA is not specific for any organ and is also produced after irradiation of the tumor. Thus, any tissue toxicity is identified using cfDNA. Rapidly responding tumors will also spill their DNA into the plasma, leading to a potentially false risk estimate. Additionally, prostate cancer was not treated with chemotherapy in this study; hence, the impact of chemotherapy on cfDNA remains another confounder of uncertain impact on the predictive value. Finally, our sample size did not allow for subset analysis of potential toxicity confounders such as prostate gland size and quantitative clinical treatment volume. To better understand the various potential confounders affecting the test, and to identify alternative uses of cfDNA, additional designs and larger studies are indicated.

In conclusion, our results suggest that plasma cfDNA levels are a promising and available test that might be used as a minimally invasive individualized predictive biomarker for acute and late patient-reported GI RT toxicity. Additional tests are underway to evaluate confounders (such as age, gender, trauma/surgery, burns, autoimmune disease, chemotherapy) and for cancers with a higher proportion of grade 3+ toxicity than prostate cancer.
